# Analysing responsible innovation along a value chain—A single‐cell protein case study

**DOI:** 10.1049/enb2.12031

**Published:** 2024-03-20

**Authors:** Monica Hoyos Flight, Joyce Tait, Theo Chronopoulos, Monica Betancor, Pauline Wischhusen, Emily Burton, Helen Masey O'Neill, Kim van der Heul, John Hays, Peter Rowe

**Affiliations:** ^1^ Innogen Institute The University of Edinburgh High School Yards Edinburgh UK; ^2^ Drax Power Station Selby UK; ^3^ Institute of Aquaculture University of Stirling Stirling UK; ^4^ Norwegian Institute of Marine Research (IMR) Bergen Norway; ^5^ Nottingham Trent University Poultry Research Unit Southwell UK; ^6^ AB Agri Lynch Wood Peterborough UK; ^7^ Deep Branch Biotechnology Ltd Nottingham UK

**Keywords:** Bio‐economy, industry, innovation, responsible research innovation, standard

## Abstract

The British Standards Institution's Publicly Available Specification 440 (PAS 440) provides a Responsible Innovation Framework (RIF) that companies can use to continuously monitor the societal, environmental and health benefits and risks of their innovations, as well as relevant changes to the supply chain and regulations. PAS 440 is intended to help companies achieve the benefits of innovation in a timely manner and avoid any potential harm or unintended misuse of a new product, process or service. Here, the authors have applied the PAS 440 RIF to a novel single‐cell protein (SCP) animal feed ingredient taking into consideration the perspectives of the value chain partners (VCPs), companies and laboratories involved in an Innovate UK research project. The authors’ findings show how VCPs can use PAS440 to demonstrate that they are innovating responsibly. Using this approach to responsible innovation along the value chain—from manufacturing scale‐up, through regulatory approval, to incorporation in animal feed and from there to food on supermarket shelves—can support the development of innovations that contribute to the economic and environmental sustainability of the animal feed sector. The authors conclude that the PAS 440 Guide can facilitate the progress of a new product throughout a value chain and contribute to coordinating responsible behaviour among companies involved in the value chain.

## Graphical abstract

The authors demonstrate the application of the British Standards Institution's PAS 440 Responsible Innovation (RI) Guide to the commercial development of a single cell protein product for use in animal feed. PAS 440 is intended to help companies achieve the benefits of innovation in a timely manner and avoid any potential harm or unintended misuse of a new product, process or service. The case study reported here shows how PAS 440 can be used by companies to demonstrate that they are innovating responsibly and how using this approach to responsible innovation can support the development of innovations that contribute to the economic and environmental sustainability of value chain partners.

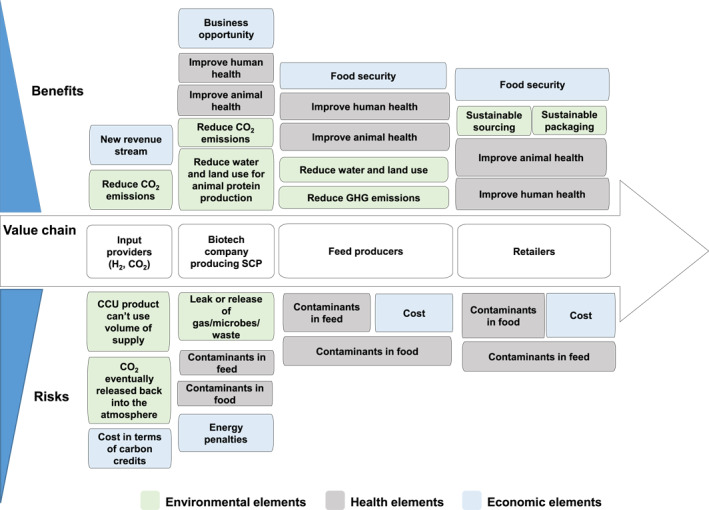

## 1 INTRODUCTION

### 1.1 Responsible innovation background

Responsible behaviour or corporate social responsibility is becoming an essential aspect of business operations in all companies, regardless of the extent of innovation involved in their business models. The ISO 26,000 Standard, ‘Guidance on social responsibility’, launched in 2010, has addressed this requirement through a set of general principles including human rights, labour practices, the environment, fair operating practices, consumer issues, and community involvement and development [1]. The EU's approach to Responsible Research and Innovation (RRI), like the ISO standard, focuses on general company‐level principles, including engagement, gender equality, science education, open access, ethics, and governance [2, 3]. A related set of developments on responsible innovation per se (RI) has specified additional responsible behaviour requirements for companies and academic innovators engaged in developing innovative products and processes. The UK's approach to RI was initially led by the Engineering and Physical Sciences Research Council whose AREA (Anticipate, Reflect, Engage, and Act) framework focused on the tools and techniques needed to deliver RI [4]. However, these RI initiatives addressed both research and innovation without making any significant distinctions between them. The BSI PAS 440 standard builds on the AREA framework and addresses the additional responsibility requirements related to the properties of the innovation as it progresses through the development process towards market availability.

This study builds on our previous work [5, 6] demonstrating that RI needs to address additional innovation‐related requirements that are not dealt with under social responsibility headings. As specified in the PAS 440 Guide, the implementation of RI by companies should consider both (a) the requirement to abide by agreed standards of societally responsible behaviour (**company level responsibility**); and (b) the specific properties of an innovative product, process or service, as it becomes part of established or new value chains, often changing company ownership in the process (**technology‐specific responsibility**) [7].

Company‐level responsibility (e.g. human rights, labour practices, consumer issues, gender equality) should be stable and uniform throughout all areas of company activity. Technology‐specific responsibility on the other hand makes additional demands. Its needs will vary depending on the nature of the innovative product, process or service, so a company will need to have a tailored approach to each innovation in its portfolio.

The first attempt to apply RI principles to UK publicly funded translational research was in 2012. A Technology Strategy Board (TSB) funding call for synthetic biology required all applicants to demonstrate how they would “… ***anticipate and give responsible consideration*** to the ***intended*** and ***potential unintended*** impacts of the commercial development and use of the technology, including the potential for misuse, ***before the work begins*”** (TSB emphases). This took the novel step of adding technology‐specific elements to the company‐specific elements of the ISO and EU social responsibility approaches. That experience contributed to later developments in RI [6] leading eventually to the publication of the Innovate UK‐funded BSI Publicly Available Specification (PAS 440) Guidance on Responsible Innovation that specifically recognised the need for company‐level and technology‐specific considerations as well as the need for a whole value chain approach [8].

PAS 440 provides a Responsible Innovation Framework (RIF) that companies can use to guide their own responsible behaviour and to demonstrate that they are innovating responsibly, charting how they identify, evaluate, record, and communicate the expected benefits and possible risks of their innovative developments. The factors that innovators are guided to consider will help them to achieve the benefits of innovation in a timely manner and avoid any potential harm or unintended misuse of a new product, process or service. This in turn will make companies more resilient, save costs, improve their sustainability, and gain customer and investor trust.

PAS 440 is intended to strike a balance between the Precautionary Principle [9] and the Innovation Principle [10] to bring safe and beneficial products to market without stifling innovation. As specified in the PAS, it is intended to be manageable by small companies with limited resources, easily incorporated into project management and risk management standard operating procedures, and to provide guidance on conducting stakeholder engagement in potentially contentious circumstances. By encouraging early engagement with stakeholders, including value chain partners (VCPs), PAS 440 can facilitate the progress of a new product throughout a value chain and contribute to coordinating responsible behaviour among companies involved in the value chain [11, 12].

### 1.2 The case study

This study is based on a translational research project, REACT‐FIRST [13], that is developing a single‐cell protein (SCP) product as an alternative feed ingredient for fish and chickens, using carbon dioxide (CO_2_) and hydrogen (H_2_) as feedstocks for microbial fermentation. In 2019, it was estimated that 10% of the UK's total GHG emissions came from agriculture, with the production of animal feed being a major contributor because of the land use and input requirements [14]. The production of SCP using captured CO_2_ could help reduce carbon emissions and thus tackle the climate emergency, and contribute to sustainable food production.

The value chain partners (VCPs) involved in the case study are part of the REACT‐FIRST consortium, which is led by the company developing and manufacturing the SCP (SCP producer), and include a power station providing the CO_2_ for the fermentation process; two feed testing facilities to ensure the SCP meets the required nutritional standards for animal feed; two animal feed manufacturers producing feed for farmed chickens and salmon; and a large supermarket chain. This project offered an opportunity to study the implementation of RI, considering the current perspectives and fore‐sighting future perspectives of different companies and of animal feed standards experts as the SCP product is developed. In the context of RI, the SCP will contribute to Net Zero and biodiversity policy goals, by reducing reliance on unsustainable feed ingredients such as soya meal and wild‐caught fish [15].

PAS 440 was previously trialled in two early stage biotechnology companies [16]: MiAlgae, producing an omega 3‐rich algae‐based oil and Norfolk Plant Sciences producing genetically modified purple tomatoes with high levels of anti‐oxidants. Representatives of these companies noted the usefulness of the RIF for potentially increasing the acceptance of new products across stakeholder groups and for being better prepared for the challenges of a rapidly changing innovation ecosystem [16].

This study describes the implementation of the PAS 440 RIF for a novel SCP feed ingredient with the objectives: (a) to trial PAS 440s guidance, identifying the social, environmental and health‐related benefits and risks of the SCP, along with regulatory elements and value chain elements; and (b) to consider the implementation of this approach to RI along the value chain, from manufacturing scale‐up, through regulatory approval, to incorporation in animal feed and from there to food on supermarket shelves. The RIF is one of the key elements of PAS 440, designed to help companies continuously monitor the societal, environmental and health benefits and risks of their innovations, as well as relevant changes to the supply chain and regulations.

Implementing the PAS 440 RIF (Table 1) along the value chain can help VCPs to be more strategically aligned so they can support the development of innovations that will have a long‐term impact on the economic and environmental sustainability of the value chain and its products. A holistic understanding of RI among VCPs, and how it changes over time, is expected to be important for future RI guidance and standard development. The PAS 440 RIF could also be a useful addition to value chain analysis toolkits for identifying more sustainable value chains and opportunities to improve climate change resilience [17].

**TABLE 1:** BSI Responsible Innovation Framework template (PAS440, section 7). Reproduced with permission from BSI.

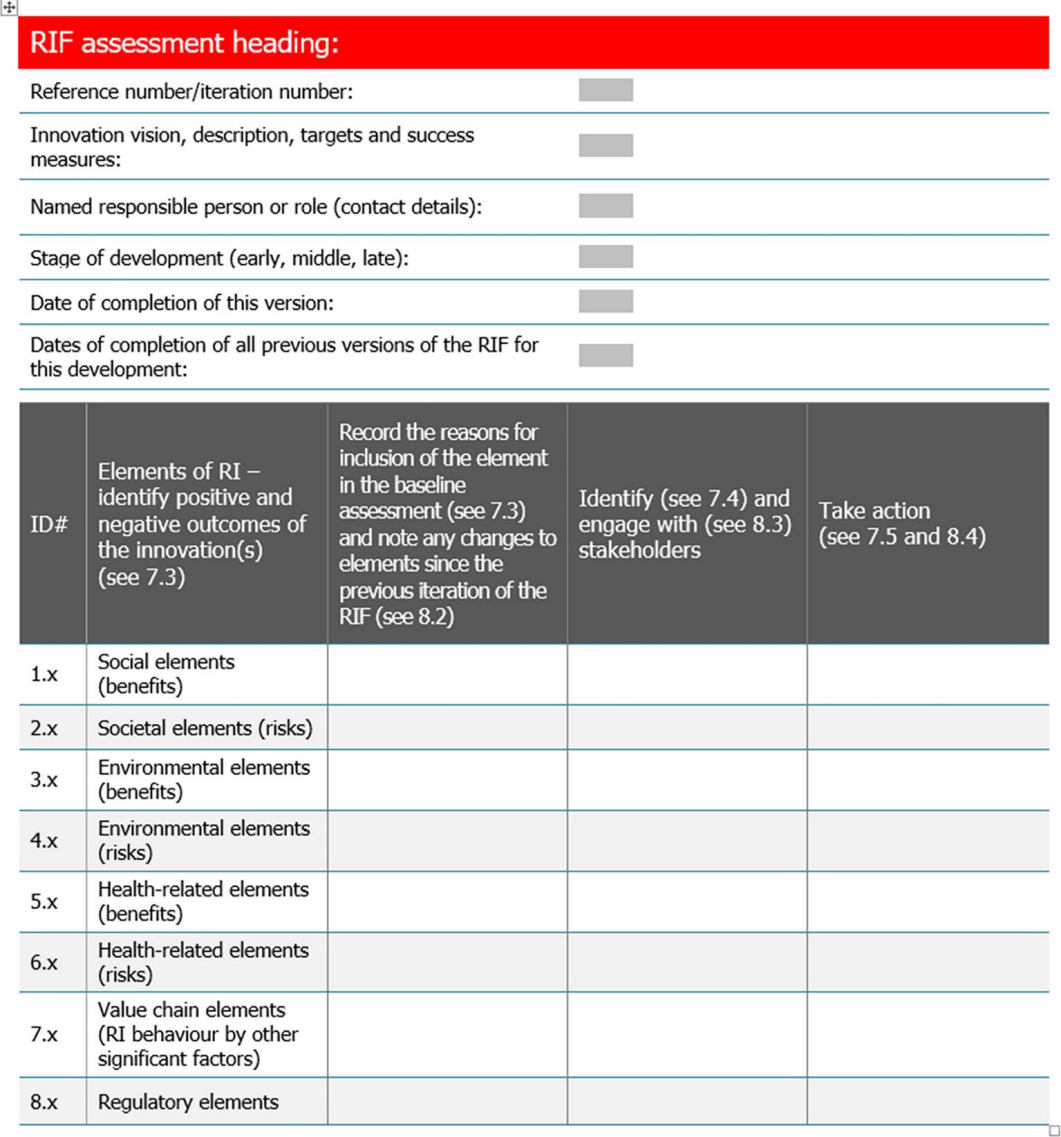

*Note*: The #ID row of the table refers to numbered sections in the PAS 440 Guide.

## 2 METHODS

This study charts the process of implementing a RI approach by the company developing the innovative product. This is an example of a product developed with the intention to contribute responsibly to Net Zero and biodiversity policy goals as well as being economically viable but which could face potential challenges due to public concerns about the use of bacteria as food.

PAS 440 provides a Responsible Innovation Framework (RIF) (Table 1) that companies can use to chart their own progress in ensuring that they are conducting innovation responsibly and to demonstrate this to others, standardising how they identify, evaluate, record, and communicate the benefits and potential risks, ensuring that no unacceptable risks are entailed in the final marketed product [8]. The factors that they are guided to consider will help them to achieve the benefits of an innovation in a timely manner and avoid any potential harm or unintended misuse of a new product, process or service. As specified in the PAS, demonstrating responsible innovation through the RIF is a means to make companies more resilient, save costs, improve their sustainability, and gain customer and investor trust.

**FIGURE 1:**
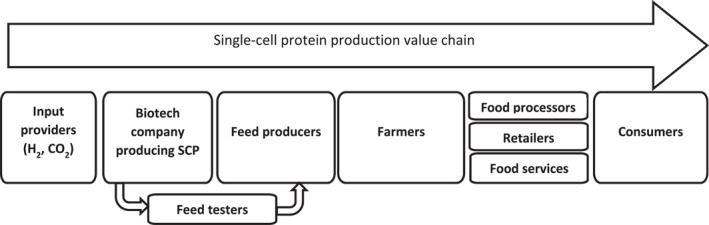
The single‐cell protein (SCP) product value chain.

The main focus of this study is on the SCP product and the company developing it, considering how PAS 440 guidance can support responsible innovation in relation to that product. VCPs participating in the project, from supplying the CO_2_ feedstock and manufacturing scale‐up (SCP producer), through regulatory approval, to incorporation in animal feed and from there to food on supermarket shelves, were considered for this RI analysis as stakeholders of the SCP producer (Figure 1). Involving a supermarket company in the project allowed us to complete the value chain through to marketing to the final consumer and enabled us to demonstrate the important role of companies at the end of a value chain in influencing all the others at early and intermediate stages of development, particularly in the context of RI. Our analysis was not able to include the perspectives of farmers, food processors and food service providers, who are also involved in the same value chain, and we did not engage directly with consumers and NGOs as stakeholders, although consumer views were surveyed by the supermarket chain.

The PAS 440 RIF template in Table 1 consists of a series of headings covering the societal, environmental and health elements of RI (positive and negative), the value chain elements (noting responsible behaviour elements for VCPs) and relevant regulations (in this case meeting feed nutritional quality standards). For this research project, members of the research team (Hoyos Flight and Tait) conducted the RIF analyses, based on interviews with staff in the company developing the SCP, with those ensuring that the product meets relevant feed quality standards and with staff in VCPs.

Interviews with the SCP producer and the VCPs were tailored in each case to the role and contribution of the interviewee. Questions were open‐ended and designed to explore the perspective of that organisation on their role in the product development and in the overall value chain and also the issues they saw as either hindering or supporting that development.

For the raw material provider, the focus was on the CO_2_ feedstock for SCP production; for the animal feed companies, the focus was on the final feed formulation, which would include a proportion of SCP, and for the supermarket, the focus was on the final chicken or fish product which would be reared on feed that includes a proportion of SCP. Thus, as the SCP moves along its projected future value chain, involving different companies (Figure 1), and being transformed into different products, the nature of each company's involvement with the product and the elements to be considered in a RI analysis will change. For the organisations testing the nutritional quality of the feed, attention was focused on the SCP itself and how it would perform as part of a feed formulation. The use of carbon dioxide and hydrogen as basic inputs for the SCP production process is considered beneficial, provided that the hydrogen is produced using renewable energy sources in that it is an additional means of carbon capture and does not rely on food‐related products such as sugars as the carbon source in the fermentation.

We first carried out a baseline RI assessment, engaging with the company developing the SCP, then a follow‐up assessment involving two rounds of interviews with the company developing the SCP, selected VCPs and the organisations undertaking dietary testing of the product for use as an animal feed. Representatives of each VCP involved in this project were interviewed twice, at an interval of approximately 1 year, to explore what RI would mean for them, as participants in a future value chain for a product similar to the SCP being developed in this case.

Built into all versions of the RI process is an emphasis on the need to engage with citizens and their representatives as stakeholders in the development of an innovative product (PAS 440, Sections 7.4 and 8.3). A public engagement initiative was beyond the resources of this study, but the research team was able to include in the analysis the results of a market research survey on public willingness to purchase food products based on animals reared on SCP ingredients that was conducted by the supermarket involved in this project as a VCP.

The RIF template (Table 1) (PAS 440, Section 7) was seen by interviewees as a useful tool for collecting and organising relevant data and for keeping track of changes over time, but it was less suitable for explaining the relevant issues to others with an interest in the outcomes who had not been involved in the analysis. We therefore developed a diagrammatic approach (see Results section) to facilitate communication among members of the research team and the wider stakeholder community.

## 3 SCP PRODUCER—BASELINE RI ASSESSMENT

The baseline RI assessment for the SCP innovation, developed in consultation with the biotechnology company, identified the societal, environmental and health‐related elements to be included in the first two columns of the RIF (Table 2). The last two columns of the RIF (Table 1), ‘identify and engage with stakeholders’ and ‘take action’, were dealt with in the analysis following the completion of the baseline assessment. The company's objectives and motivations behind the development of the SCP product were strongly influenced by its ambitions to contribute to today's environmental, health and societal challenges, making a strong positive business case based on the RI dimension. However, RI also requires a company to demonstrate awareness of any associated risks or ethical concerns.

**TABLE 2:** SCP production company RIF based on table 1‐ Baseline Assessment Date of completion of this version: 24^th^ July 2021.

	Elements of RI—positive and negative outcomes	Reasons for inclusion
Societal elements (benefits)	Contribute to a circular economy and economic growth	Improve the acceptability of animal protein production and meet Net Zero policy ambitions
	Improve food security—meet the increased demand for fish and chicken protein	Address growing concerns about food security and global supply chains
Societal elements (risks)	Inability to source raw materials cost‐effectively	Negative impact on economic viability of the value chain
	Poor uptake by VCPs	Negative impact on economic viability of the value chain and lack of social acceptance
	High cost of drying the product for formulation in feed	Negative impact on the overall economic viability of SCP product
Environmental elements (benefits)	Reduce the environmental impact of animal protein production	Reduce GHG emissions of chicken and salmon farming, protect biodiversity by reducing reliance on wild fisheries and soya for feed production, and reduce use of land and water for feed/food production
Environmental elements (risks)	Plant/facility explosion due to hydrogen gas leakage	Need to meet demanding safety regulations
	Leakage of viable micro‐organisms	Important regulatory standards involved
	Energy required for drying the product	Increase in the carbon footprint of the SCP product
Health elements (benefits)	Fish and chickens are healthier dietary options than red meat	Increase consumption of fish and chicken
Health elements (risks)	Contaminants in feed arising from the production process	Need to meet demanding feed standards and potential impact on product acceptability in the food chain

As part of the baseline assessment, we identified important stakeholders for the company developing the SCP product. The views of these stakeholders will inform future actions to realise the opportunities and mitigate the risks of the SCP production and point to ways to demonstrate RI. Table 3 lists the stakeholders we identified as relevant to this RI analysis and highlights those included among the VCPs with whom we were able to engage directly. We were not able to engage directly with ‘wider society’ stakeholders, but the supermarket, being closest to consumers in the value chain, undertook a survey of customers to gauge their attitudes to inclusion of novel ingredients in fish and chicken feed. In the policy and governance‐related category, we engaged directly with the feed testing facilities in Stirling and Nottingham Trent Universities.

**TABLE 3:** SCP manufacture—Stakeholders identified^a^.

Value chain and related partners	Policy and governance‐related	Wider society
*Raw material providers*	Regulators	Advocacy groups/NGOs
*Feed producers*	Policy makers	Citizens
Farmers	Politicians	Consumers
*Supermarkets*	*Feed testing facilities*	Press/media
Industry lobby groups		

^a^ Those in italics are included among VCPs for this project.

The baseline assessment, summarised in Tables 2 and 3, was the starting point for follow‐up interviews in the second and third years of the project with the SCP company, with VCP partners, and with feed testing facilities.

## 4 FOLLOW‐UP INTERVIEWS AND FULL RI ASSESSMENT

### 4.1 SCP producer

**FIGURE 2:**
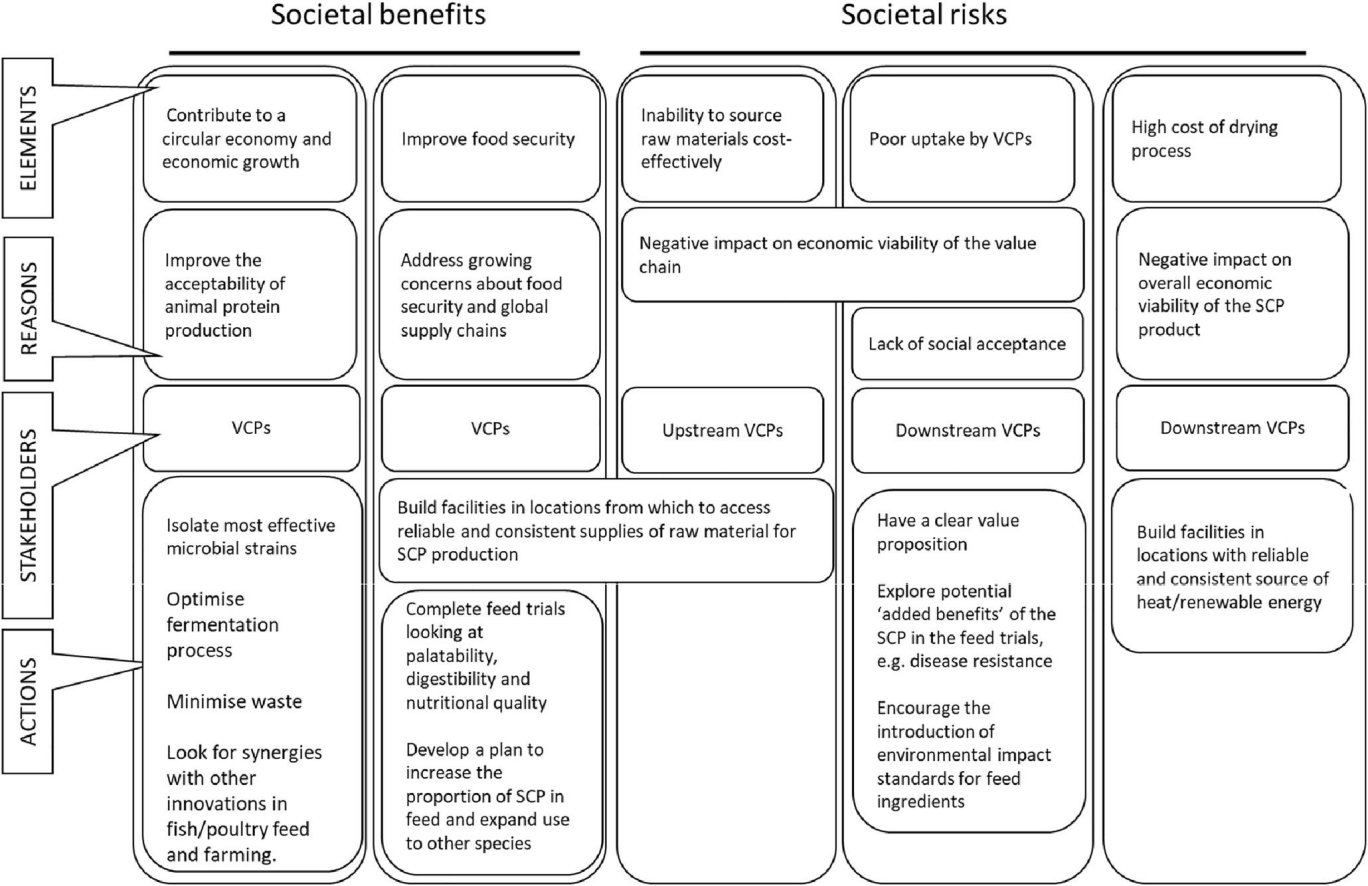
Follow‐up assessment, SCP production company RIF—societal elements.

**FIGURE 3:**
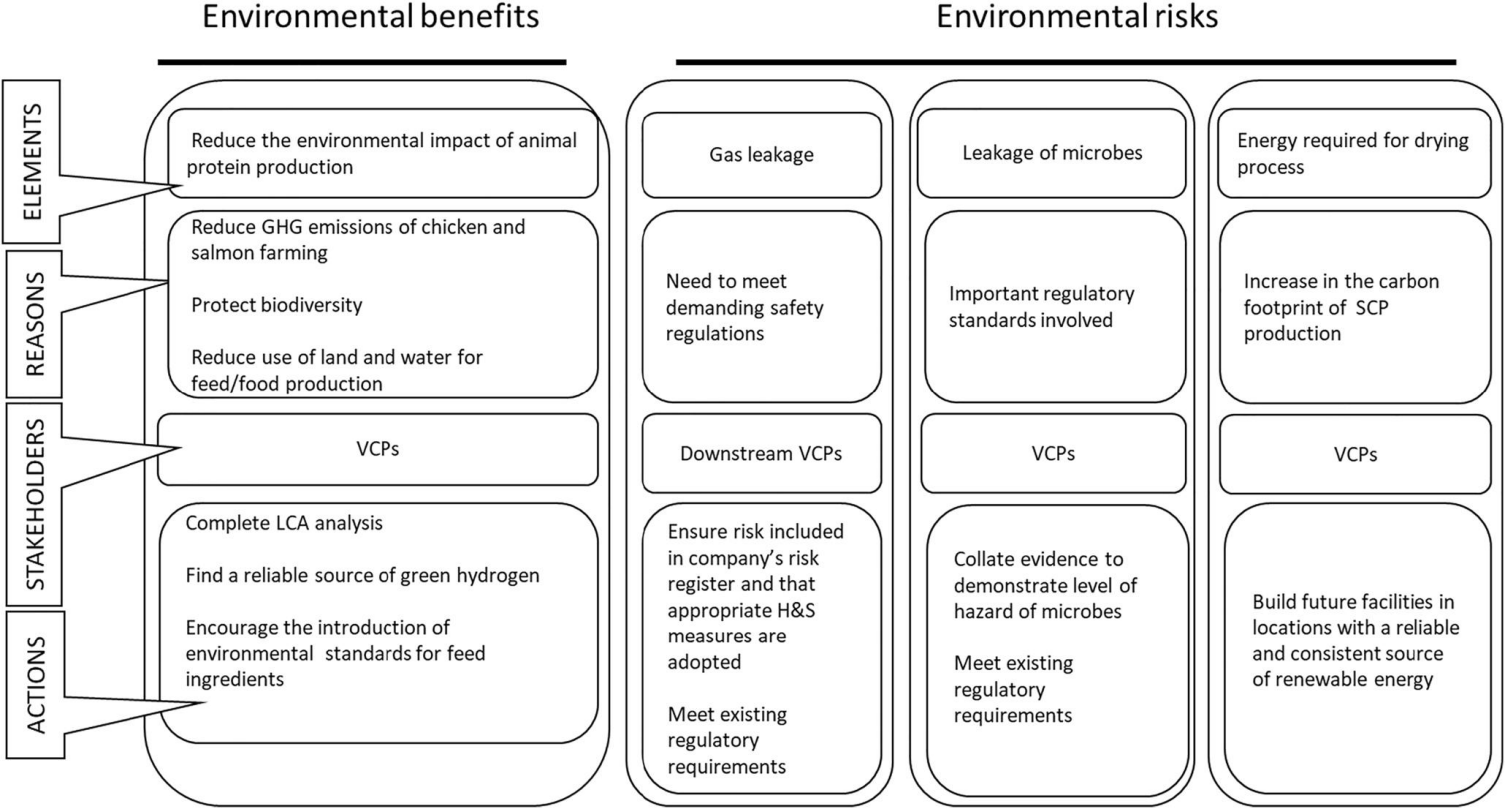
Follow‐up assessment, SCP production company RIF—environmental elements.

**FIGURE 4:**
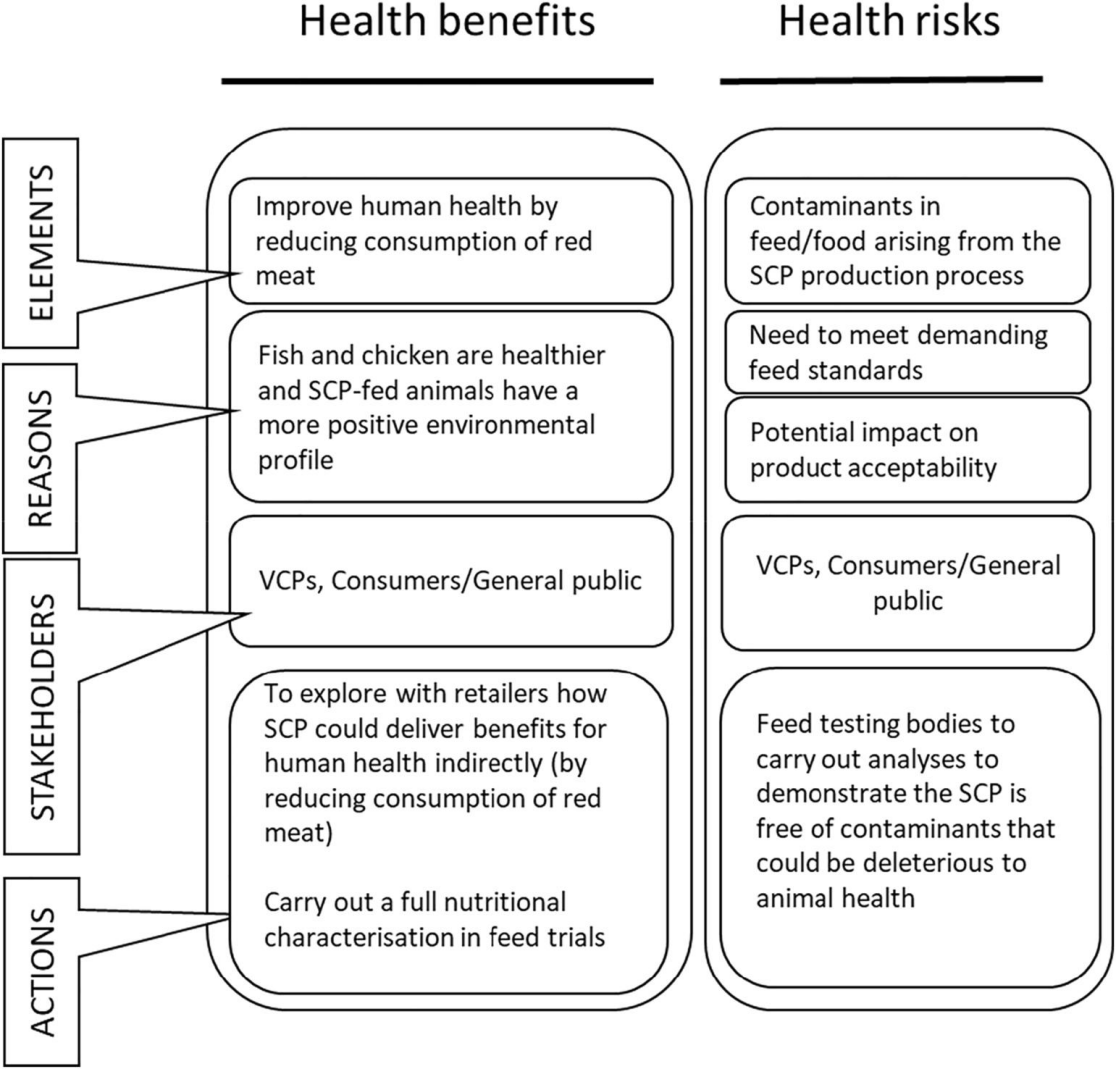
Follow‐up assessment, SCP production company RIF—health‐related elements.

Figures 2, 3, 4 illustrate all aspects of the RIF (Table 1) relevant to the company developing the SCP. These figures build on the baseline assessment in Table 2 and include the stakeholders we engaged with and actions to be taken.

There are overlaps in the categories to which risks and benefits are assigned. Some benefits or risks can be seen as both societal and environmental, or both societal and health‐related, and where such overlaps occur, we have included the element in both categories.

The actions suggested in the RIF to manage the benefits and mitigate the risks of the SCP product can help the company reduce costs in the long‐term and lead to the development of a more resilient product that is more attractive to investors, VCPs, customers, and society. Staff at the company agreed that this project has improved supply chain relationships, increased awareness of VCPs' concerns, and improved the SCP producer's innovation capabilities and long‐term planning.

#### 4.1.1 Societal benefits and risks

The intended societal benefits of the SCP include contributing to an economic system based on the reuse and regeneration of materials or products, known as the circular economy [18], by supporting recycling of waste materials (CO_2_ in this case), and improving food security. To achieve these benefits, company actions (summarised in the bottom half of Figure 2) include isolating the most efficient bacterial strains for SCP production, optimising the fermentation process, minimising waste production and looking for synergies with other innovations in fish or poultry feed and farming (for example, novel feed additives to promote animal health being produced by other companies or potentially by the SCP producer).

Additional benefits could be achieved by using engineering biology to increase the level of protein output from the fermentation process and/or to improve its nutritional quality by tailoring its amino acid composition to the specific requirements of different animal species. For example, as explained by the animal feed testing facilities in this study, the ideal amino acid composition of protein in animal feed varies across different species [19]. However, the SCP producer was reluctant to become formally involved in engineering biology, the only economically feasible way to achieve this aim, given the nervousness about the use of these technologies by other companies closer to consumer markets.

Pursuit of these additional benefits by the company will depend on the success of the feed trials being conducted by the feed testing facilities. If successful, further trials would be needed to evaluate the possibility of increasing the proportion of SCP in feed and of incorporating it into the feed of other species.

The societal risks discussed in interviews included difficulties in responsibly sourcing starting materials (CO_2_ and H_2_ that are not derived from processes involving fossil fuels); the high costs (financial and energy‐related) of drying the SCP product for incorporation into feed pellets; and potential poor uptake of SCP by animal feed producers and farmers. The company planned to mitigate these risks by locating future facilities near reliable, consistent and responsible sources of CO_2_ and H_2_, as well as affordable, renewable energy sources (such as geothermal) for the drying process. When sourcing CO_2_, the company plans to seek suppliers that can meet the volume and purity requirements for food and feed‐related production.

#### 4.1.2 Environmental benefits and risks

Reducing the environmental impact of animal protein production, while maintaining economic viability, was the main RI‐related benefit of the innovative product. By replacing some of the soya meal currently in use in animal feed, the producer expects the SCP to reduce the GHG emissions of chicken and salmon farming, reduce biodiversity loss, and lower the amount of land and water used in feed and food production (Figure 3). Actions identified as being necessary for the delivery of these benefits included: finding a source of green hydrogen produced without the use of fossil fuels or electricity derived from fossil fuels. The company was also committed to developing a life cycle analysis (LCA) to quantify the environmental benefits and costs arising from the production and use of the SCP product and to contribute to the introduction of environmental standards for feed ingredients.

Risk‐related elements included hydrogen gas leakage from the facility being built to manufacture the SCP. This proved to be the most significant relevant risk to materialise during the course of the project. The scale‐up facility being built to manufacture the SCP did not initially meet the required safety standards for dealing with the explosive risks of H_2_ use, causing significant delays. Among other more serious difficulties, this meant that the RI analysis is not as rich as it would have been if the project had progressed on the planned timescale.

Another potential risk would be leakage of viable micro‐organisms during the production process. This is minimised by regulations and standards with which the company is expected to comply. The high energy cost associated with drying the fermentation product was noted under societal considerations and, in a RI context, the source of this energy would need to be environmentally sustainable.

#### 4.1.3 Health‐related benefits and risks

Realising the potential health benefits of the SCP for animals and (indirectly) humans will require compliance with current food and feed safety standards to address any risks that may have been introduced through the SCP product (Figure 4). As part of this project, feed trials are being carried out to demonstrate the safety of the product and to ensure that the SCP is free of contaminants that could be deleterious to animal health.

### 4.2 VCP perspectives on the SCP and RI

Interviews with VCPs provided insights into their expected future involvement with SCP products, what business models they would adopt and what responsibility‐related factors would be relevant to them. The results are interpreted in terms of their implications for the company developing the SCP and how this would influence their future engagement with that particular VCP (Figure 1). However, the VCP companies are all large organisations involved in many different value chains, and so for them, engagement with this value chain has only a marginal impact on their overall business model. Interestingly, the companies we worked with did not see RI as a constraint but quite the opposite, as a way of ensuring future gains.

#### 4.2.1 CO_2_ input provider

In the early stages of SCP production, CO_2_ was sourced from a power station generating electricity from biomass (wood pellets). The company aims to become carbon negative by 2030 by deploying next‐generation technologies that can capture carbon in the long term with high efficiency and low cost (carbon capture and storage (CCS)).

The company has also been considering technologies such as SCP production that involve carbon capture and use (CCU), ultimately releasing CO_2_ back into the atmosphere, but avoiding the use of fossil fuels in its manufacturing process [20]. The company hosts an incubation area where other companies and start‐up businesses are testing carbon capture (CC) technologies under real conditions, using flue gas from the combustion of biomass. Given the scale of operations for a power station, any CCU option would need to be able to commit to using a continuous stream of CO_2_ (approx. eight million tonnes per year) for 10–20 years.

The company's commitment to RI helps to attract collaborators and research partners with the shared ambition of reducing CO_2_ emissions from the energy sector. It also employs dedicated teams that focus on engaging with the local community and stakeholders at a national level to assess how the company can further reduce its carbon footprint.

In making decisions about future collaborations and investments, the company is influenced by UK Government policy to prioritise public investment in CCS over CCU [21], although they still see an important role for biogenic CO_2_ as a process feedstock in a future Net Zero economy.

From the perspective of the company developing the SCP, there are also good reasons to move to another source of CO_2_ that better matches the scale and purity they require.

#### 4.2.2 Chicken feed producer

The company describes itself as a nutrition company selling compound feed as well as individual ingredients and additives. In the RI context, their internal drive is to ‘do the right thing’ and produce feed that has the least possible impact on the planet, and this aim is shared with other partners in the various value chains with which they are involved. They also noted a shift in the nature of conversations with clients encouraging supermarkets to become more interested in shaping the market towards environmental sustainability.

Incorporating the SCP into feed formulations has the potential to meet environmental, societal and health objectives, but the main driver when formulating nutritionally appropriate feed has always been to minimise the cost in this very competitive sector. Any new protein source will be competing with existing inputs, soya being the market leader, and SCP incorporation would increase the price of feed and food. Another factor contributing to this equation is how the deforestation associated with land use for soya production is accounted for. After 20 years (around 2028), the associated carbon cost of soya will drop [22], meaning that alternatives will need to meet the newly reduced carbon vales of soya to be competitive on a carbon basis (as well as price).

Because of the volatility of the market and seasonality of agricultural commodities, feed producers see value in the SCP's potential to improve food security by ensuring a reliable and consistent supply of protein. However, progress in the replacement of current feed ingredients is still expected to be slow, requiring a whole‐industry approach to reduce the carbon footprint of feed using a variety of measures supported by government policy initiatives. Companies developing innovative feed ingredients may find it easier to break the cost barrier by modifying organisms to produce an ingredient with a defined amino acid content so feed mixes can be tailored to the nutritional requirements of animals and minimise waste (‘precision feed’).

As part of their RI strategy to move away from soy and reduce their carbon footprint, the chicken feed producer is exploring various new feed ingredients, including other SCPs and micro‐algae. They are also encouraging RI throughout their own value chains, with responsibility codes of conduct for their suppliers, and working with customers on the design of feed trials with novel ingredients. They observed that companies at the end of the value chain have the greatest leverage to influence those nearer the beginning, and that change is most likely to happen when supermarkets signal the unacceptability of a particular element in the food chain. Such tipping points can also be supported by government policy, and others in the supply chain need to be prepared for that.

#### 4.2.3 Salmon feed producer

This company's approach to RI was similar to that of the chicken feed producer, with a focus on reducing carbon emissions and minimising land use, within the constraints of financial viability and competition with other companies. They are already using 5% SCP in fish feed in Norway, and they expect to incorporate up to 10% in future, partly enabling them to move away from deforested soya (they are currently buying 50% of the market volume of European (non‐deforested) soya). The source of H_2_ to produce the SCP will also need to be carbon neutral. As for the chicken feed producer, they conduct their own life cycle analyses (LCA), but they do not publish the results, although they do report on emissions reduction targets through the Science Based Targets initiative [23]. They aim to be producing feeds that are 50% circular and restorative (including mitigating land use change and biodiversity loss) by 2030 and consider SCP as a key contributor to that goal. Through their sustainability team, they are also engaging with stakeholders across the value chains they are involved in to make sustainability the main driver of innovation in the fish farming industry.

The company would favour the use of genetic technology to develop future innovative feeds, for example, to modify the micro‐organism to change the amino‐acid profile of the SCP product or to increase pigmentation (a value added), but there is too much uncertainty from consumer, retailer and legislative perspectives. There are also several competing SCP products in development along with insect and worm‐based feed. All these technologies are facing scale‐up and cost issues and could also be limited by fermentation feedstock requirements, as they will face competition from biofuel/biogas production. Government initiatives will probably be needed to support the scale‐up of novel feed ingredients.

#### 4.2.4 Supermarket chain

The supermarket chain involved in this project aims to reduce the carbon footprint of its supply chain and its environmental impact while meeting customer needs for safe, healthy and affordable food. It considers RI to be part of its business strategy, although it does not use this term, and is working proactively with VCPs to encourage innovation to meet its sustainability goals, rather than waiting for consumers to demand change. A cross‐value chain approach to RI that deepens the understanding of the strategic priorities of VCPs is seen as important when it comes to innovation and can help support the translation of innovative products from proof‐of‐concept to the market.

The SCP could contribute to meeting the retailer's aims by lowering the carbon footprint of salmon and chicken farming and reducing its impact on biodiversity, an issue that NGOs and the media have brought to customers' attention. This has not yet translated into consumer demand for alternative feed products, but the public stakeholder environment has changed since 2020 when the project began, and consumers are more accepting of such innovations. Even if the SCP only makes up 5%–10% of the feed, the supermarket would consider it as a significant contribution to reducing the carbon footprint of farmed protein.

A survey carried out by the supermarket suggested that consumers may be willing to pay more for products that improve animal welfare and contribute to protecting biodiversity and that they are highly accepting of the concept of environmentally friendly feeds. However, experience shows that purchasing decisions are mainly made on quality and price.

Although the supermarket chain cannot make contracted suppliers use particular feeds, they could require them in their own‐branded produce where they fully own the supply chain. There is also an increasing trend for vertically integrated farmers to make their own feed, to a specification provided by the retailer, rather than purchasing it from a feed manufacturer. However, given the price sensitivity of chicken and salmon products, retailers would need an incentive or subsidy to do so and all retailers would need to move at the same time, as is already happening with the big four supermarkets in the UK signing up to use deforestation‐free soya. This retailer is wary of using positive labelling as an incentive to purchase such products, and any labelling would probably focus on the outcome, organic or low carbon and not on how it was achieved.

#### 4.2.5 Value chain summary perspective

**FIGURE 5:**
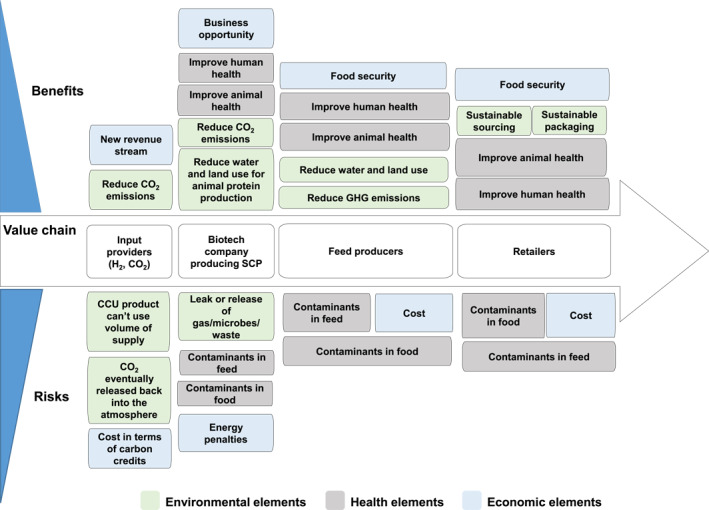
VCP perspectives on RI‐related risks and benefits of the production and use of the SCP. *Note*. *Items closest to the central line were most important to the company.

The different perspectives of VCPs on the approach to RI in the production and use of the SCP are summarised in Figure 5. The core value chain is similar to that illustrated in Figure 1, and the perceived benefits and risks for each VCP are listed above and below this value chain, with the most important being placed closest to the central line. This innovative diagram is a useful way of summarising and explaining the results of the RI analysis developed using the RIF (Tables 1 and 2), as explored in the Discussion and Conclusions section. By looking at how the relevant elements change, or do not change, as the product moves along the value chain, it highlights how the same elements can be perceived as benefits by more than one of the partners, for example, reduce water and land use for animal protein production. In any LCA, it will be important to decide to which company these benefits should be allocated and to ensure that there is no double counting across the value chain.

This summary diagram may have made the companies involved in the project more aware of the perspectives of their VCPs, although it was notable that VCPs were already well aware of the perspectives of others in the value chain. However, as a case study, it serves to raise awareness about PAS 440 and may encourage others to develop their own RIF to help guide conversations around the responsible development and adoption of innovations.

### 4.3 Animal nutritionists' perspectives

Two feed testing facilities were involved in the project to test the safety, quality and acceptability of the SCP for Atlantic salmon and chicken feed, including nutritional value, health impacts and palatability of feed containing up to 15% SCP. Because high levels of nucleic acid in SCP in feed can interfere with protein metabolism and cause health problems in longer‐lived animals [24], this percentage is likely to be limiting on the eventual contribution of the SCP to Net Zero animal protein production, even if its cost can be brought down. An additional processing stage can be introduced to reduce the nucleic acid content, but this would add to the cost‐related disincentives for adoption.

The legal requirements for the conduct of feed trials in the UK include safeguarding animal and human health and animal welfare, environmental impact, the use of additives and the use of genetic technologies (GM) in feed production [25]. It is common practice, as a minimum, to carry out a full nutritional characterisation and test digestibility and palatability. Further tests include examining macro and micro structural changes to the digestive systems (e.g. by lesion scoring and histological analyses) and assessing welfare indicators (e.g. signs of discomfort or negative impacts of excreta on the litter or water quality).

A potential added benefit of a novel feed could be improving disease resistance in the farmed species, which can be tested for by challenging the fish or poultry with specific pathogens or assessing established markers of intestinal health. For GM technologies, testing is required to demonstrate the absence of introduced genes, for example, in fish that have been fed omega‐3 oils from GM plants.

The testing laboratories also noted that some companies have their own specific testing criteria beyond those that are legally required, that can change depending on media interest, for example, sustainability and responsibly sourced ingredients, problems with terrestrially sourced plant based feed, and growing customer interest in animal welfare.

Balancing the amino acid content is important for feed producers and they will consult the testing laboratories on the need to add amino acid feed supplements to create the ideal formulation. Becoming a producer of a high value feed supplement, such as omega‐3 fatty acids, rather than of the commodity protein element of feed, was suggested as an easier market for the SCP provider to penetrate.

## 5 DISCUSSION AND CONCLUSIONS

### 5.1 RI analysis at company and value chain levels

This case study explored how a small company developing an innovative food‐related SCP product demonstrated a commitment to responsible innovation, guided by the PAS 440 framework. SCP production is one of the innovative developments using industrial biotechnology to substitute for more polluting or environmentally damaging technologies that can contribute to mitigate climate change, protect biodiversity, and improve food security.

The case study also trialled the process of involving business partners throughout the overall product value chain (VCPs) in demonstrating compliance with PAS 440 guidance. It considered how and where differences would emerge in the interpretation of RI as the SCP product was taken up by other companies along the value chain and translated into protein‐rich foods on the supermarket shelf (Figure 5). The companies included in this project are interacting in relation to RI‐related issues in a way that would not exist as part of normal business dealings, so this cannot be seen as a test of how RI might be implemented in practice. In the absence of the stimulus of the translational research grant in which all SCPs were involved as partners, an alternative arrangement would need to be found as a stimulus for collaboration across an emerging value chain and as a basis for collating and integrating the results.

However, the case study does illustrate the potential benefits of creating circumstances where RI could be formally integrated along a value chain. Value chain studies have highlighted the importance of forming collaborative relationships and sharing information to improve the economic performance of companies [26, 27]. Our study suggests that adopting a RI approach along a value chain can help VCPs to be more strategically aligned and support the translation of innovative products from proof‐of‐concept to market. Translational research funders could helpfully make it standard practice to include this element in future funding calls.

The PAS 440 RIF was used to facilitate conversations with VCPs on the development of an SCP product. For companies that are already having conversations around innovation with VCPs, the PAS 440 RIF could be useful for structuring or formalising these discussions. Advantages of the PAS440 RIF include that it is designed for companies of any size and sector, it can be carried out with limited time and staff investment since it focuses on exploiting existing knowledge rather than on new research, and it can be implemented on a product‐by‐product basis. As described, in the case of the SCP, it contributes to focussing ‘new’ research on the most important problems.

For the SCP producer, solving environmental and health challenges facing society is part of the company's ethos, and these aspirations are included in the RIF as benefits. Likewise, threats to the successful economic development of the product could be seen as RI‐related risks, delaying or preventing achievement of the expected benefits.

Our findings suggest that integrating a RI approach across companies in the value chain would contribute to identify consumer and VCP requirements, foresee future benefits and risks, and adapt the development of new products or technologies accordingly. Long‐term engagement among VCPs with shared values will help to create a market for this and similar SCP products [28] by raising wider awareness of the environmental and health issues around animal feed, helping consumers to make sustainable food choices and encouraging the introduction of environment‐related standards for animal feed ingredients in compliance, for example, with the UN sustainable development goals [29].

The VCPs in this project were interested in improving their environmental performance by using responsibly sourced raw materials or feed products. For the CO_2_ input provider, there are potential economic and environmental benefits, while for downstream VCPs, using the SCP could aid the environmentally sustainable production of healthy feed and food. The main environmental concern in the companies we engaged with was carbon emissions, although some had also set biodiversity‐related goals, guided by the 2030 Agenda for Sustainable Development [30]. VCPs downstream of the SCP producer also saw SCP as potentially providing greater security of supply of raw materials for food and feed value chains, and this will be a selling point for companies developing this type of product, provided they can overcome scale‐up and cost‐related issues.

A point that came out clearly from our analysis is that **the company at the end of the value chain**, with a business‐to‐consumer business model, has the greatest power to influence all other value chain participants; upstream VCPs need to be prepared to foresee and react to changing demands from downstream companies. By working together, upstream VCPs will be guided to focus their efforts on innovations that are best suited to meet the demands of downstream VCPs. Through these collaborations, they may also be able to identify synergies with other innovations to maintain or enhance productivity and potentially reduce costs, along with opportunities to create more circular or networked value chains that contribute to reducing waste [31].

The survey conducted by the supermarket chain sent a positive message back along the value chain about the likely consumer reception for more environmentally sustainable animal feeds, but also a warning about the need to avoid all but minor increases in cost. The higher cost of food products based on SCP animal feed would restrict their adoption to higher priced niche products with only a small proportion of the total market. In the short term, the limited market would limit the scale of the overall environmental benefit, although this could also ease the way for future wider uptake if public support for such initiatives is maintained and increased.

An alternative approach to product marketing that does not involve large retailers and could extend the options available to SCP producers and feed companies could become more influential in future. It involves locally organised value chains that can be financially viable on a small scale and can contribute to waste reduction and circular economy developments [31]. One of the feed companies involved in this project is working to create markets for innovative feed products by fostering long‐term partnerships with customers, developing smaller, locally supplied feed and food chains to avoid or minimise waste through circular economy initiatives.

The **feed producers in the middle of the value chain**, buying products from and selling products to other business, are influenced by their customers, farmers whose feed purchasing decisions in turn will often be determined by contracts with a supermarket chain. Where only niche, high value, markets are envisaged in the short term, as explained above, the immediate demand for SCP feed ingredients will be limited. Thus, in addition to the 5%–10% limit on SCP incorporation in feed for economic reasons and because of its nucleic acid content, the short term SCP‐containing feed may only be used in a small proportion of the fish or chicken products on the market. These factors should be noted in any life cycle analysis used to calculate the contribution of SCP to meeting overall Net Zero policy commitments.

Feed producers will also be monitoring the RI credentials of suppliers manufacturing ingredients to be used in their animal feeds and will want to be able to point to responsible sourcing of ingredients used in earlier stages of production such as SCP manufacture.

**SCP producers at the beginning of the value chain** will be particularly aware of the need for responsibly sourced manufacturing inputs, and the suppliers of these inputs will be aware of similar pressures from other potential customers. These pressures are also often transmitted back along the value chain, starting from the final consumer. The supplier of CO_2_ for SCP manufacture was involved in this project, but not the H_2_ supplier, and it is particularly important to have a H_2_ source that does not involve the use of electricity generated from fossil fuels. The need for an appropriate source of CO_2_ and green hydrogen are key considerations for the SCP producer when deciding where to locate its future scaled‐up manufacturing sites.

Consumers today will hold companies responsible for mistakes made by partner companies at any point along a value chain [32], prompting all companies to take an interest in whether and how business partners are meeting RI standards. Within the constraints of the funded research project, this case study has illustrated how this requirement could be met and how the insights gained can provide useful insights for all companies involved in the value chain.

### 5.2 Regulatory and policy elements

Meeting the requirements of relevant regulations is an important component of RI (see Table 1), and this case study included two feed testing facilities among the project participants. This is perhaps the most important component of the regulatory environment for novel animal feed products, and in this case study, it illustrates how this element can be incorporated into a RI analysis. Discussions between the SCP producer and the testing laboratories meant that the SCP producer was better informed about the details of the regulatory requirements, allowing for earlier adaptation of their production processes where necessary, saving time and money.

The testing labs were also in a position to comment on alternative innovation options for the SCP producer. They noted that there are opportunities to develop the SCP as part of a feed mix tailored to the nutritional requirements of particular species by genetically modifying the micro‐organisms to produce protein with a specific amino‐acid content and/or to produce omega‐3 fatty acids, an essential health ingredient for salmon as part of human diets. However, although the SCP producer could have benefitted from the use of genetic technologies to improve the quality of its product in a number of ways, the amount of additional testing likely to be required in such cases would be a disincentive, along with uncertainty about future consumer perspectives on the use of genetic technologies.

A related comment from another VCP was that some life science companies may be over‐interpreting ‘responsibility’ and avoiding any links with genetic technologies, even where they can have useful benefits for health or the environment and are not subject to additional regulatory requirements.

Regulatory and policy elements play a very important role in enabling or constraining specific innovations, sometimes in ways that are disproportionate and act counter to the delivery of societal benefits and RI [33]. This case study has demonstrated how bringing the regulatory element into RI‐related considerations can play the obvious role of ensuring that companies comply with regulations designed to ensure the safety, quality and efficacy of products. However, it can also play a more proactive role in supporting RI by working with all VCPs to encourage smarter regulation and policies designed to support the delivery of more healthy, societally useful and environmentally sustainable products.

### 5.3 Implications for future RI standard development

We see this case study as a contribution to future methodological development for technology‐specific RI based on the application of the PAS 440 RIF to a novel SCP feed ingredient, leading to more general insights into the future development of RI‐related standards. The RIF table itself (Table 1) proved to be an effective tool for summarising and highlighting the key aspects of RI for this product and for enabling the SCP producer to keep track of the main elements involved. However, the table itself was not so effective at communicating these factors to other VCPs involved in the case study, prompting the use of the graphics in Figures 2, 3, 4. Further development of diagrammatic approaches to RIF presentation would be a useful addition to future RI‐related guidance, particularly in helping the company concerned to demonstrate compliance with the RI standard.

This case study explored in detail elements 7 and of the RIF (Table 1) on ‘value chain elements’ and ‘regulatory elements’, respectively. Both are important components of the innovation ecosystem for the development of an innovative product, and they could be interpreted in the RI context in a routine ‘tick‐box’ fashion. However, this project explored in more detail how they could be applied pro‐actively, using the baseline information from interviews and discussions to enable the SCP producer to be more creative in shaping the future properties of the product and its innovation ecosystem. From the perspective of regulatory elements, this would include both ensuring compliance with the existing regulatory system and engaging with regulators and other stakeholders to guide future changes in governance systems to cover novel properties of the product or adaptations needed to existing regulations or standards. The whole value chain approach (Figure 5) enabled the companies involved in the project to understand better the properties of the SCP and its role in their business models, to appraise its contribution to their own RI‐related agendas, and to appreciate more clearly how this area of innovative animal feed development can be better supported in future.

As the importance of responsible innovation becomes more widely recognised, across a broad range of industry sectors and across nation states, evidence of compliance with an RI standard will become an increasingly valuable asset for companies. This case study contributes to future thinking about such developments, considering how the PAS 440 approach can be implemented and how it can usefully be adapted and/or complemented by bringing in additional concepts and considerations, particularly those related to managing and improving the translational ecosystem for innovative technologies.

We anticipate that, as the PAS 440 RIF gains wider recognition for promoting the acceptance of new products throughout their value chain and bringing safe and beneficial products to market, it could develop into an accreditable British Standard, or part of an International Standard.

## AUTHOR CONTRIBUTIONS

**Monica Hoyos Flight**: Conceptualisation; Data curation; Formal analysis; Methodology; Project administration; Validation; Writing – original draft; Writing – review & editing. **Joyce Tait**: Conceptualisation; Formal analysis; Funding acquisition; Methodology; Supervision; Writing – original draft; Writing – review & editing. **Theo Chronopoulos**: Formal analysis; Investigation; Resources; Writing – review & editing. **Monica Betancor**: Formal analysis; Investigation; Resources; Writing – review & editing. **Pauline Wischhusen**: Formal analysis; Investigation; Resources; Writing – review & editing. **Emily Burton**: Formal analysis; Investigation; Resources; Writing – review & editing. **Helen Masey O'Neil**: Formal analysis; Investigation; Resources; Writing – review & editing. **Kim van der Heul**: Writing – review & editing. **John Hays**: Formal analysis; Investigation; Resources; Writing – review & editing. **Peter Rowe**: Conceptualisation; Funding acquisition; Investigation; Resources; Writing – review & editing

## CONFLICT OF INTEREST STATEMENT

The authors declare no conflicts of interest.

## PERMISSION TO REPRODUCE MATERIALS FROM OTHER SOURCES

BSI granted permission to reproduce Table 1 from the PAS 440.

## Data Availability

Data sharing not applicable—no new data generated.
